# Titin and diaphragm dysfunction in mechanically ventilated rats

**DOI:** 10.1007/s00134-012-2504-5

**Published:** 2012-02-11

**Authors:** Hieronymus W. H. van Hees, Willem-Jan M. Schellekens, Gilberto L. Andrade Acuña, Marianne Linkels, Theo Hafmans, Coen A. C. Ottenheijm, Henk L. Granzier, Gert-Jan Scheffer, Johannes G. van der Hoeven, P. N. Richard Dekhuijzen, Leo M. A. Heunks

**Affiliations:** 1 Department of Pulmonary Diseases, Radboud University Nijmegen Medical Centre, Nijmegen, The Netherlands; 2 Department of Anesthesiology, Radboud University Nijmegen Medical Centre, Nijmegen, The Netherlands; 3 Department of Intensive Care Medicine (631), Radboud University Nijmegen Medical Centre, P.O. Box 9101, 6500 HB Nijmegen, The Netherlands; 4 Department of Molecular and Cellular Biology, University of Arizona, Tucson, AZ USA

**Keywords:** Mechanical ventilation, Diaphragm, Force, Single fiber, Myosin, Titin

## Abstract

**Purpose:**

Diaphragm weakness induced by mechanical ventilation may contribute to difficult weaning from the ventilator. For optimal force generation the muscle proteins myosin and titin are indispensable. The present study investigated if myosin and titin loss or dysfunction are involved in mechanical ventilation-induced diaphragm weakness.

**Methods:**

Male Wistar rats were either assigned to a control group (*n* = 10) or submitted to 18 h of mechanical ventilation (MV, *n* = 10). At the end of the experiment, diaphragm and soleus muscle were excised for functional and biochemical analysis.

**Results:**

Maximal specific active force generation of muscle fibers isolated from the diaphragm of MV rats was lower than controls (128 ± 9 vs. 165 ± 13 mN/mm^2^, *p* = 0.02) and was accompanied by a proportional reduction of myosin heavy chain concentration in these fibers. Passive force generation upon stretch was significantly reduced in diaphragm fibers from MV rats by ca. 35%. Yet, titin content was not significantly different between control and MV diaphragm. In vitro pre-incubation with phosphatase-1 decreased passive force generation upon stretch in diaphragm fibers from control, but not from MV rats. Mechanical ventilation did not affect active or passive force generation in the soleus muscle.

**Conclusions:**

Mechanical ventilation leads to impaired diaphragm fiber active force-generating capacity and passive force generation upon stretch. Loss of myosin contributes to reduced active force generation, whereas reduced passive force generation is likely to result from a decreased phosphorylation status of titin. These impairments were not discernable in the soleus muscle of 18 h mechanically ventilated rats.

**Electronic supplementary material:**

The online version of this article (doi:10.1007/s00134-012-2504-5) contains supplementary material, which is available to authorized users.

## Introduction

Mechanical ventilation is a life-saving intervention in critically ill patients, but comes with several adverse events including weakness of the diaphragm muscle [1, 2]. This is clinically important as weakness of the inspiratory muscles plays an important role in difficult weaning from mechanical ventilation [3–5]. Several studies have indicated that respiratory muscle weakness induced by mechanical ventilation primarily results from changes within the diaphragm muscle fibers. For example, diaphragm fibers isolated from patients and rodents that underwent controlled mechanical ventilation show decreased cross-sectional areas, i.e. atrophy [6–8], which most likely affects the force-generating capacity of the diaphragm. Moreover, studies on isolated diaphragm bundles from mechanically ventilated animals demonstrate reduced generation of specific force, i.e., force per cross-sectional area [9–12]. This either suggests that contractile protein concentration is reduced or that remaining contractile proteins have reduced functionality. Measuring contractile function of permeabilized muscle single fibers is the most appropriate technique to study the involvement of contractile protein dysfunction [13]. Force-generating capacity of permeabilized muscle fibers strongly depends on the content of the contractile protein myosin [14]. Accordingly, the first aim of the present study was to investigate whether reduced force-generating capacity of diaphragm muscle fibers from mechanically ventilated rats is associated with decreased myosin concentration.

In addition to atrophy, signs of muscle fiber injury like myofibrillar disarray and Z-band streaming have been observed in the diaphragm of mechanically ventilated humans and animals [8, 9, 15, 16]. The mechanisms leading to muscle fiber injury are largely unknown. For structural stability and optimal active force generation, passive elastic structures are indispensable [17]. Titin is the major determinant of passive elastic properties of striated muscle [18]. In an animal model of peripheral muscle disuse a preferential loss of titin explained abnormal sarcomeric organization [19]. Passive elastic properties of skeletal muscle fibers can also be modulated by posttranslational modifications or alternative splicing of titin [20], as occurs in diaphragm fibers from patients with COPD [21]. The effect of mechanical ventilation on titin function in respiratory muscles is unknown. Accordingly, the second aim of the present study was to investigate the passive elastic properties of permeabilized diaphragm fibers from mechanically ventilated rats.

Previous studies indicated that the diaphragm is more susceptible to the deleterious effects of mechanical ventilation than peripheral skeletal muscles [6, 7, 10]. Whether the effects of mechanical ventilation on muscle protein function are different between respiratory and peripheral muscle is currently unknown. Therefore, we additionally determined active and passive force generation of soleus muscle fibers from the same animals. We hypothesized that mechanical ventilation induces loss of myosin and titin, resulting in reduced active and passive force generation of diaphragm muscle fibers. We expected the impairments to be less prominent in soleus muscle fibers.

## Methods

### Experimental design

Two groups of male Wistar rats (body weight ca. 300 g) were studied, controls (*n* = 10) and after 18 h of mechanical ventilation (MV, *n* = 10). Five to six isolated skinned single muscle fibers from the diaphragm and the soleus of each animal were investigated in terms of cross-sectional area, myosin heavy chain isoform and concentration, calcium-induced maximal active force generation and passive force generation upon stretch (total number of approximately 60 fibers per group). Titin content was studied in diaphragm homogenates and in diaphragm cryosections. Since impaired passive force generation upon stretch in diaphragm fibers of MV rats was not accompanied by reduced titin content, we subsequently examined the effect of posttranslational dephosphorylation on passive force generation of the rat diaphragm. All experiments were approved by the Regional Animal Ethics Committee (Nijmegen, the Netherlands) and performed under the guidelines of the Dutch Council for Animal Care.

### Animal model

Our animal model of mechanical ventilation was based on previously described methods [11], with minor modifications as described in detail in the “Electronic supplementary material” (ESM).

### Skinned fiber contractile measurements

Maximal active force generation and passive force generation upon stretch of skinned single fibers isolated from the diaphragm muscle were determined as described previously [22] (see ESM for details).

### Effect of posttranslational dephosphorylation

The effects of posttranslational dephosphorylation on passive force generation were investigated according to previous described methods [23]. Skinned diaphragm bundle preparations were incubated with phosphatase-1 (PP-1; 1.5 U/μl) in relaxing solution for 2 h at room temperature. Subsequently single fibers were isolated and passive tension upon stretch was measured as described in the ESM.

### Myosin heavy chain isoform and concentration

Determination of myosin heavy chain isoform composition and content by SDS-PAGE was described previously [24] and adapted from Geiger et al. [14]. See ESM for details. Since only five diaphragm fibers from each group expressed the slow isoform of myosin heavy chain, these were excluded from further analysis. Accordingly all fibers were classified as type II.

### Titin content

Content of the major sarcomeric protein titin was determined by SDS-agarose gel electrophoresis and by immunohistochemistry as described previously [21]. See ESM for more details.

### Statistical methods

The sample size of approximately 50 fibers per group, calculated a priori, was based upon the assumption of detecting a reduction of passive force of at least 25%, with a standard deviation of 50% [21], 80% probability, and an alpha level of 0.05. Differences between MV and control rats regarding maximal force generation, myosin heavy chain concentration, and titin content were analyzed with Student’s *t* tests. Repeated measures analysis was performed with post hoc Student* t* testing at each fiber length to evaluate the statistical significance of differences in single fiber passive tension data between groups. A probability level of *p* less than 0.05 was considered significant. Mean ± SE values are presented in text, tables, and figures.

## Results

### Animal characteristics

MV rats exhibited stable hemodynamics during 18 h mechanical ventilation (mean arterial pressure 99 ± 1 mmHg) and pH, PaO_2_, and PaCO_2_ were normal at the end of the experiment (7.48 ± 0.02, 13.4 ± 0.8 kPa, and 5.5 ± 0.2 kPa, respectively).

### Active force generation

**Fig. 1:**
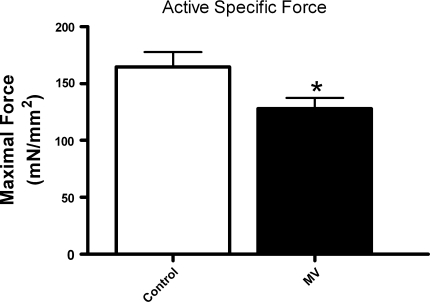
Maximal active specific force generation of diaphragm fibers from control and mechanically ventilated rats (*MV*). *p < 0.05

The cross-sectional area of skinned diaphragm fibers from MV animals was ca. 25% lower than in control animals (2.0 ± 0.1 vs. 2.6 ± 0.2 × 10^−3^ mm^2^, *p* = 0.002), which is consistent with the development of muscle fiber atrophy (see ESM Fig. 2). Moreover, mechanical ventilation significantly reduced diaphragm fiber force-generating capacity, even when corrected for loss of cross-sectional area (*p* = 0.02, Fig. 1).

In soleus muscle fibers mechanical ventilation affected neither the cross-sectional area (2.4 ± 0.1 vs. 2.6 ± 0.2 × 10^−3^ mm^2^, for MV and control rats) nor the specific force-generating capacity (136 ± 6 vs. 127 ± 12 mN/mm^2^ for control and MV rats).

### Myosin heavy chain concentration and active force

**Fig. 2:**
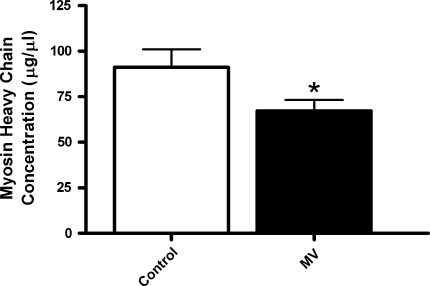
Myosin heavy chain concentration in diaphragm fibers from control and mechanically ventilated rats (*MV*). *p < 0.05

Myosin heavy chain concentration was ca. 25% lower in diaphragm fibers from MV animals than in diaphragm fibers from control animals (*p* = 0.03, Fig. 2). Maximal force generation per myosin molecule, i.e., maximal force divided by myosin heavy chain content, was not significantly different between MV diaphragm fibers and control (2.31 ± 0.26 and 2.97 ± 0.55 N/mg respectively, *p* = 0.3).

Myosin heavy chain concentration in soleus muscle fibers was not significantly affected by mechanical ventilation (80 ± 12 vs. 79 ± 10 μg/μl for control and MV animals, respectively).

### Passive force generation

**Fig. 3:**
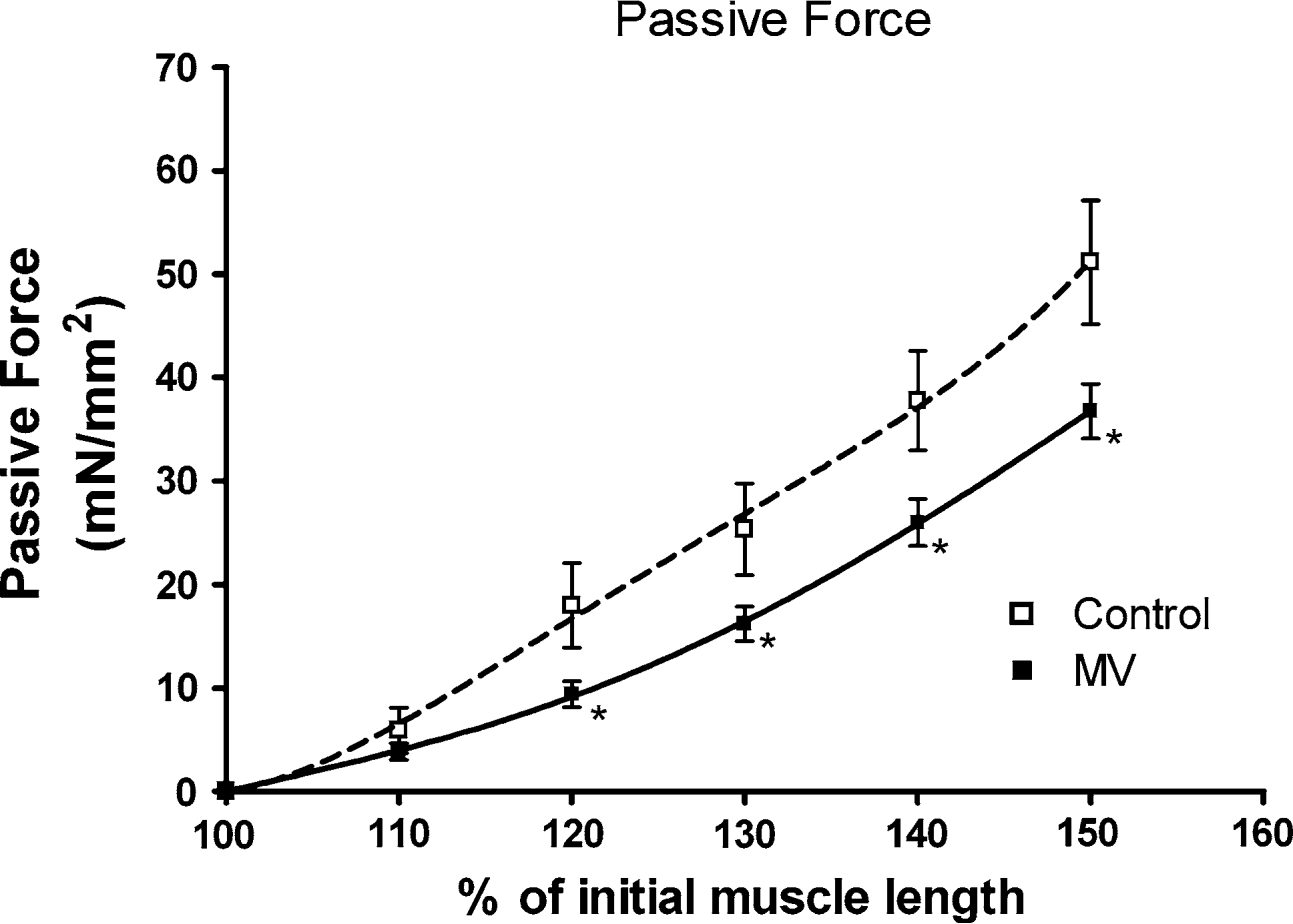
Passive force generation at different muscle fiber lengths of diaphragm fibers from control and mechanically ventilated rats (*MV*). *Lines* represent fourth-order polynomial fits. *p < 0.05

Passive force generation upon stretch was impaired in diaphragm fibers from MV animals (Fig. 3). At fiber lengths longer than 120% of optimal length, diaphragm fibers from MV animals displayed significantly lower passive forces compared to fibers from control rats.

In soleus muscle fibers passive force generation was not affected by mechanical ventilation. For example, at 140% of optimal fiber length soleus fibers generated passive forces of 47 ± 3 versus 51 ± 4 mN/mm^2^ for control and MV animals respectively.

### Titin content

Reduced passive tension of skeletal muscle fibers can result from loss of titin, increased expression of longer titin molecules, or posttranslational modifications of titin [20].

**Fig. 4:**
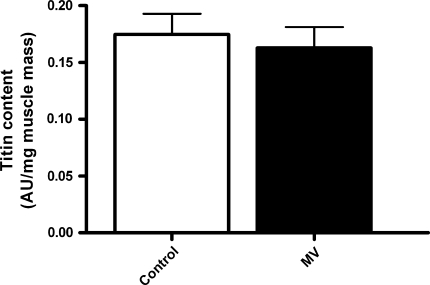
Titin content in diaphragm samples from control and mechanically ventilated rats (*MV*)

Titin content analyzed by SDS-agarose gel electrophoresis was not significantly different in diaphragm samples from MV animals compared to control (Fig. 4). Furthermore, the content of titin’s degradation product T2 in the diaphragm was not significantly different between MV and control rats (2.7 ± 0.5 vs. 4.0 ± 0.9 AU/μg muscle weight, *p* = 0.23), which excludes titin breakdown as a reason for lower passive force in MV fibers. To gain insight into the size of titin in diaphragm muscle of MV rats, we studied the mobility of titin on gel. A representative gel (ESM Fig. 3) shows that the mobility of titin from rat diaphragm samples is distinctly faster than the mobility of titin from human soleus muscle, which is explained by a smaller molecule size. The mobility of titin was not different between diaphragm samples from MV rats and controls, indicating the absence of detectable size differences in titin.

**Fig. 5:**
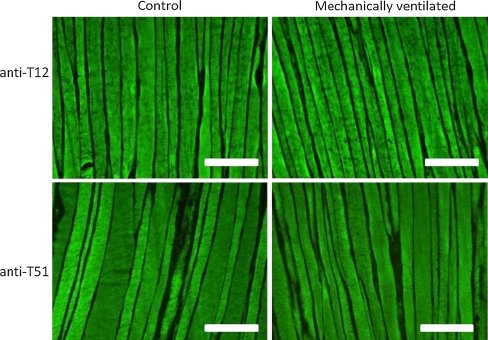
Representative photographs of cryosections of the diaphragm from control and mechanically ventilated rats stained with antibodies against two specific titin epitopes T12 (Z-line) and T51 (M-line). *Bar* 100 μm

Immunohistochemical analysis confirmed the absence of titin loss in the diaphragm of MV rats. Staining intensities of antibodies directed against the titin epitopes T12 (Z-line) and T51 (M-line) were comparable between MV and control diaphragm (Fig. 5).

### Passive force and dephosphorylation

**Fig. 6:**
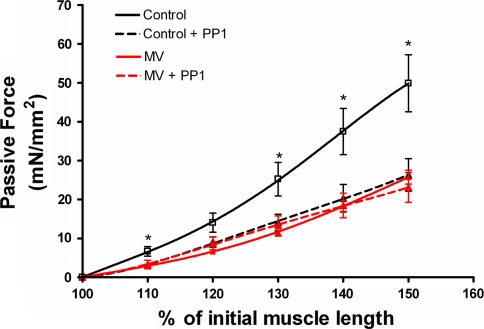
Effect of dephosphorylation on passive force generation at different muscle fiber lengths of diaphragm fibers from control and mechanically ventilated rats (*n* = 5 fibers per group). *Lines* represent fourth-order polynomial fits. *Dotted lines* fibers were pre-incubated with phosphatase-1 (PP-1, 1.5 U/μl, for 2 h). *p < 0.05 versus control +PP1

As titin content and size were not affected by mechanical ventilation, we investigated if posttranslational modification of titin could explain the increased compliance of diaphragm fibers in MV animals. Recent studies have shown that titin’s compliance can be increased by lowering the phosphorylation state of titin [23]. Accordingly, we examined the effect of dephosphorylation on passive force generation of diaphragm fibers from control and MV rats. Figure 6 shows that pre-incubation with PP-1, a dephosphorylating enzyme, significantly reduced passive force generation upon stretch in diaphragm fibers from control animals. However, PP-1 did not affect passive force generation in diaphragm fibers of MV rats.

## Discussion

Previous studies have reported that controlled mechanical ventilation has profound effects on the structure and force-generating capacity of the inspiratory muscle in humans and animal models [1, 6–8, 10, 12]. The data of the present study provide the following new and important insights into the effects of 18 h controlled mechanical ventilation on diaphragm muscle function: (1) in addition to atrophy, decreased myosin concentration contributes to reduced active force generation, (2) mechanical ventilation significantly reduces passive force of diaphragm muscle fibers, (3) the effects of mechanical ventilation on passive force can be mimicked by dephosphorylating the elastic protein titin, and (4) the effects of mechanical ventilation on active and passive force do not occur in the soleus muscle within this time frame.

### Reduced force generation and loss of myosin

Several studies, both in humans and in animal models, showed that mechanical ventilation rapidly induces atrophy of diaphragm muscle fibers [1, 6–8]. Our results are in line with those studies as we found that 18 h mechanical ventilation provoked a 20% reduction of diaphragm fiber cross-sectional area. The capacity of a muscle to generate force strongly depends on the number of crossbridges that can be formed in parallel [14]. As such, reduction of diaphragm muscle fiber cross-sectional area may explain reduced diaphragm force output when cross-sectional area and contractile protein content decrease proportionally. Yet, reduction of cross-sectional area only partially explains mechanical ventilation-induced diaphragm weakness, because several studies have shown that prolonged mechanical ventilation reduces diaphragm bundle or single fiber specific force-generating capacity, i.e., absolute force divided by cross-sectional area [9–12, 25]. Our data are in line with these studies and show that fiber specific force-generating capacity is already reduced after 18 h of mechanical ventilation (Fig. 1). Moreover, we found that myosin concentration is reduced in diaphragm fibers from mechanically ventilated animals (Fig. 2), indicating that the severity of myosin loss exceeds the degree of fiber atrophy. This can explain the large decrease in contractile protein content recently observed in the diaphragm of mechanically ventilated humans [26]. Notably, after correction of force for myosin concentration, no differences exist in force between control and ventilated diaphragm. This implies that contractile protein loss is a strong determinant of diaphragm weakness upon mechanical ventilation. In addition, this finding indicates that the remaining contractile proteins display normal maximal force-generating capacity. This is in contrast to the diaphragm in patients with COPD, where loss of force results from reduction in protein content and impaired function of the remaining contractile protein [27, 28]. Contractile protein loss can result from both increased proteolysis and reduced synthesis. Indeed, previous studies have demonstrated that synthesis of contractile proteins is reduced [29], and proteolytic systems like the ubiquitin–proteasome pathway, caspase-3, calpains, and lysosomes are activated in the diaphragm of mechanically ventilated rodents and humans [6, 7, 12, 26, 30, 31].

### Titin function modulation due to mechanical ventilation: causes and consequences

Passive elastic structures within muscle are indispensable for structural stability and optimal active force generation [17]. Titin is the most important determinant of passive elastic properties of striated muscle fibers [18]. The current study demonstrates that mechanical ventilation reduces the stiffness of diaphragm muscle fibers. As disuse may enhance titin degradation [19], we anticipated that reduced stiffness of diaphragm fibers from mechanically ventilated animals resulted from the loss of titin. Surprisingly, both agarose gel electrophoresis and histochemical analysis demonstrated that titin content in diaphragm fibers is not affected by mechanical ventilation. Alternatively, reduced elasticity of muscle fibers can result from posttranslational modifications of titin [32]. The part of titin that largely determines its elastic properties is the PEVK (Pro-Glu-Val-Lys) segment, which encodes a coil-like structure that acts as a molecular spring [33]. Accordingly, posttranslational modifications of amino acids within the PEVK segment may modulate the stiffness of the titin molecule. The present findings indicate that titin’s stiffness depends on its phosphorylation state, because incubation with the dephosphorylating enzyme phosphatase-1 (PP-1) reduces the stiffness of diaphragm fibers from control animals. This is in line with previous findings in cardiac muscle, which showed that phosphorylation of two constitutively expressed sites within the PEVK segment increased the stiffness of isolated cardiomyoctyes [23]. In contrast to control diaphragm fibers, PP-1 did not affect the elastic properties of diaphragm fibers from mechanically ventilated rats. These data suggest that the phosphorylation state of titin’s PEVK domain in diaphragm fibers from mechanically ventilated animals is already low. Therefore, we conclude that mechanical ventilation decreases the phosphorylation status of titin in diaphragm fibers, resulting in reduced stiffness. Phosphorylation of specific sites in the PEVK domain is controlled by protein kinase C (PKC) [23]. Interestingly, a recent study showed that 3 days of disuse leads to a rapid reduction of PKC activity in peripheral skeletal muscles [34]. Since the diaphragm is contractile inactive during mechanical ventilation, it may be hypothesized that mechanical ventilation reduces PKC activity in the diaphragm.

Alterations in diaphragm muscle stiffness as reported in our study may be linked to muscle protein degradation and synthesis. Proteolytic systems can be activated by the abundance of damaged proteins and indeed, evaluation by electron microscopy revealed sarcomeric injuries in diaphragm fibers of mechanically ventilated humans and animals [8, 9, 15, 16]. Elastic structures inside and outside striated muscle fibers provide sarcomeric stability and minimize the occurrence of sarcomeric injuries. As titin is the main elastic protein within the sarcomere, deletion of titin is known to result in a loss of muscle fiber elasticity and sarcomeric injuries [17]. The loss of stiffness in the diaphragm fibers of ventilated rats may contribute to the occurrence of sarcomeric injuries and promote the degradation of contractile proteins, such as myosin. A second consequence of reduced titin stiffness is that it may affect muscle protein synthesis. Evidence exists that titin can act as a mechanosensor regulating protein expression in a sarcomere strain-dependent fashion. Two studies demonstrated that reduced strain on a kinase domain of titin results in downstream inhibition of muscle protein synthesis [35, 36]. It has therefore been hypothesized that reduced stiffness of titin in the diaphragm fibers from mechanically ventilated animals leads to a lower strain on titin kinase and in this way impedes muscle protein synthesis [37]. This would make sense as reduced strain may occur when the load on the diaphragm is reduced and less muscle mass is required.

### Respiratory versus peripheral muscle

Although the respiratory and peripheral muscles are inactive during controlled mechanical ventilation, several studies have demonstrated that the diaphragm is more sensitive to the detrimental effects of mechanical ventilation than peripheral muscles. Diaphragm atrophy in rodents occurs as early as 12 h of mechanical ventilation [31], whereas it takes much longer periods of mechanical ventilation to induce significant reductions of peripheral muscle mass [7, 10]. In humans that underwent mechanical ventilation for 18–69 h, diaphragm fiber cross-sectional area reduced by more than 50%, whereas pectoralis major fibers did not show any signs of atrophy [6]. Our data are in line with those findings as we did not observe a significant reduction of soleus fiber cross-sectional area in mechanically ventilated animals. More importantly, our data show that active and passive force-generating capacities of soleus muscle fibers were not affected by mechanical ventilation. So, in contrast to the diaphragm, muscle protein function in the soleus is preserved at least up to 18 h of mechanical ventilation. We recognize that the comparison of fast-type diaphragm fibers to slow-type soleus fibers may be confounded by fiber-type-specific effects of mechanical ventilation. However, this seems unlikely, because a previous study showed that all diaphragm fiber types developed atrophy upon 18 h of mechanical ventilation [7].

### Clinical relevance

Mechanical ventilation-induced diaphragm dysfunction complicates weaning from the ventilator [3, 4]. Weaning failure is a major clinical problem, as it occurs in a large group of patients undergoing mechanical ventilation, it increases the risk of secondary complications, it prolongs rehabilitation, and it puts a huge financial burden on the healthcare system [38]. Although supported modes of ventilation may reduce the development of respiratory muscle atrophy [39], controlled mechanical ventilation is inevitable in certain patients. In fact, a recent study advocated the use of controlled mechanical ventilation in early ARDS [40]. Unfortunately, no adequate therapies are currently available that improve diaphragm function in these patients. The current findings indicate that loss of myosin and titin dephosphorylation contribute to mechanical ventilation-induced diaphragm dysfunction. The development of treatment strategies aimed at preventing or reversing myosin loss and titin dephosphorylation might therefore be an interesting focus of future studies on mechanical ventilation-induced diaphragm weakness.

## Electronic supplementary material

Below is the link to the electronic supplementary material.

Supplementary material 1 (DOC 1123 kb)
