# Supplementary material for: Titin and diaphragm dysfunction in mechanically ventilated rats
Source: Intensive Care Med. 2012 Feb 11;38(4):702–9. doi: 10.1007/s00134-012-2504-5 (PMC3308006; doi:10.1007/s00134-012-2504-5)

**Titin and diaphragm dysfunction**

**in mechanically ventilated rats**

Hieronymus W.H. van Hees, Willem-Jan M. Schellekens, Gilberto L. Andrade Acuña, Marianne Linkels, Theo Hafmans, Coen A.C. Ottenheijm, Henk L. Granzier, G.J. Scheffer, Johannes G. van der Hoeven, P.N. Richard Dekhuijzen and Leo M.A. Heunks

***Supplemental Digital Content***

*Animal model*

Experiments were carried out in male Wistar rats (Harlan, Horst, The Netherlands). Control rats were anesthetized with pentobarbital (50 mg/kg) and sacrificed without being mechanically ventilated. MV rats were anesthetized with an intra-peritoneal injection of pentobarbital (induction 50 mg/kg), orally intubated and mechanically ventilated (ventilator UB 7025 from Hugo Sachs, March-Hugstetten, Germany). An indwelling carotic artery catheter was used for continuous measurement of blood pressure and periodic blood sampling (at 0.5, 2, 4, 8, 12 and 16 hours) for analysis of arterial pH and blood gases (i-STAT, Blood Gas Analyzer, Abbot, Hoofddorp, The Netherlands). Rats were ventilated with a tidal volume of 6 ml/kg bodyweight, respiratory rate of 110/min, positive end-expiratory pressure of 1.5 cmH_2_O and inspired oxygen fraction of 0.45. During experiments rats were fed with AIN-76 rodent diet via an oro-gastric feeding tube (approximately 2.5-3.0 kcal per hour, as previously described [1]) and received a continuous intra-venous dose pentobarbital (10 mg/kg/hour) via a tail vein catheter, which resulted in complete diaphragmatic inactivity. During experiments rats received continuously ringer’s solution of 2 ml/hour intravenously and body temperature was kept at 36-37°C.

*Tissue collection*

After 18 hours of mechanical ventilation (MV group) or immediately after anesthesia (controls), rats were exsanguinated, a combined thoracotomy and laparotomy was performed and the diaphragm and soleus muscle were quickly excised. The diaphragm was divided in three parts. From one part a muscle bundle was dissected parallel to the longitudinal axis of the muscle fibers, pinned to cork and stored at 4°C in a relaxing solution, containing 50% glycerol (vol/vol). The same was done for the soleus muscle. After 24 hours the muscle strips were stored at -20°C for later analysis of single fiber contractile properties. The second part of the diaphragm was snap-frozen in liquid nitrogen cooled isopentane and stored at –80 °C for immunofluorescence microscopy. The third part of the diaphragm was quickly frozen in liquid nitrogen and stored at –80°C for titin content analysis on SDS-agarose gel.

*Skinned fiber contractile measurements*

Relaxing and activating solutions consisted of 1.0 mM MgCl_2_, 4.0 mM Na_2_ATP, 5 mM EGTA, 10 mM imidazole, 15 mM creatine phosphate and sufficient KCL to adjust the total ionic strength to 150 mM at pH 7.0. The negative logarithm of the free Ca^2+^ concentration (pCa) of the relaxing solution was ~9.0 while in activating solutions the pCa was 4.5. Approximately one hour prior to determination of single fiber contractile properties, the muscle bundle was transferred to relaxing solution (5°C) containing 1% Triton X-100 to permeabilize lipid membranes. From the muscle bundle ~2mm segments of single fibers were isolated using microforceps. Subsequently, the fiber ends were attached to aluminium foil clips, and mounted on the single fiber apparatus. Fibers were mounted in a temperature-controlled (20 °C) flow-through acrylic chamber (120-µl volume), with a glass coverslip bottom, on the stage of an inverted microscope (model IX-70; Olympus, Amsterdam, The Netherlands). Two stainless steel hooks were used to mount the fiber horizontally in the chamber. One end of the fiber was attached to a force transducer (model AE-801; SensoNor, Horten, Norway) with a resonance frequency of 10 kHz, whereas the other end was attached to a servomotor (model 308B; Aurora Scientific, Aurora, ON, Canada) with a step time of 250 µs. In relaxing solution, sarcomere length was set at 2.4 µm as the optimal fiber length for force generation [2, 3] with the use of a calibrated eyepiece micrometer. MIDAC software (Radboud University, Nijmegen, The Netherlands) and a data-acquisition board were used to record signals. Muscle fiber length (~1.0–1.5 mm) was measured using a reticule in the microscope eyepiece [×10 Olympus Plan 10, 0.30 numerical aperture (NA)]. The fiber width was measured with a ×40 objective [×40 Olympus Plan 40, 0.60 NA]. The ×40 objective also was used to measure the fiber depth by noting the displacement of the microscope’s objective while focusing on the top and bottom surfaces of the fiber. Three width and depth measurements were made along the length of the fiber, the average values were used to calculate the fiber cross sectional area, assuming that the fiber was ellipsoid in shape. To ensure stable attachments throughout the mechanical protocol, the fiber and clip-hook attachments were first exposed to high forces generated in maximal activating solution (pCa 4.5). Maximum isometric force was determined by measuring force after perfusing the experimental chamber with, successively, pCa 9.0 and pCa 4.5 solutions. Maximum specific force was derived from dividing maximum isometric force by fiber cross-sectional area. Then the fiber was kept in relaxing solution (pCa 9.0) and passive tension–length relationships were determined by applying a repeated stretch-hold protocol, as described previously [4] and adapted from Wang et al. [5]. Stretches were applied from initial fiber length, which is the length at which sarcomere length is 2.4µm. The stretch protocol was composed of stretches of 10% of initial fiber length. Between stretches, 60 s holds were applied to allow for stress relaxation. At the end of the 60 s hold, passive tension was recorded and normalized to fiber cross sectional area. Fibers were stretched 5 times to a final length of 150% of optimal fiber length. Passive tensions were plotted against relative fiber lengths, i.e. % of initial length. Passive tension at each fiber length was expressed as the difference between actual recorded passive tension and initial passive tension (at initial fiber length).

*Myosin heavy chain concentration*

Single fibers were detached from the force transducer and servo-motor and solubilized in 25 μl SDS sample buffer. Sample volumes of 8 μl were loaded on 7% SDS-polyacrylamide gels to separate proteins. Gels were silver stained according to the procedure described by Oakley et al. [6]. Myosin heavy chain isoforms were identified by comparing migration patterns with those of control rat diaphragm bundle samples run on the same gels. Since myosin heavy chain in only 5 diaphragm fibers from each group were characterized as slow, these were excluded from further analysis. After densitometer imaging (Syngene, Cambridge, UK) myosin heavy chain content in the muscle fibers was deduced from the optical densities of known contents of purified rabbit myosin heavy chain (Sigma Aldrich, Zwijndrecht, the Netherlands) run on every gel, see Figure 1 for a typical example. Myosin heavy chain content per half sarcomere, at sarcomere length of 2.4 μm, was calculated through dividing fiber myosin heavy chain content by the number of half-sarcomeres (2 × length of fiber/2.4).

*Titin content*

Frozen diaphragm samples were first weighed and then pulverized to a fine powder and solubilized at 60°C in sample buffer (1 mg of tissue per 80 μl) containing 8M Urea, 2M Thiourea, 3% SDS, 75mM DTT, 0.05M Tris-HCl, 0.03% bromophenol blue, 25% glycerol and 10 μM leupeptin, 10 μM E64 and 0.5 mM phenylmethylsulfonylfluoride (PMSF), pH adjusted to 6.8. Samples were loaded on SDS-agarose (1%) gels and electrophoresed. Gels were stained with Coomassie brilliant blue and scanned. A range of volumes (at least three) of each sample was electrophoresed on the same gel. Wet gels were scanned and analyzed with one-D scan software (Scanalytics Inc, Fairfax, VA). The integrated optical density (OD) of titin and its degradation product T2 were determined at each loading. Using regression analysis, the slope of the linear range of the relation between integrated OD and loading for each protein was calculated.

*Immunohistochemistry*

Cryosections (7μm thick) from frozen diaphragm specimens were rehydrated for 10 minutes in PBS and blocked with PBS containing 1% (w/v) BSA. Cryosections were incubated with antibodies against two epitopes of titin, one near the Z-line and one near the M-line These antibodies, respectively called T12 and T51, were a kind gift from dr. P. van der Ven (Dept of Molecular Cell Biology University of Bonn, Bonn, Germany). Then cryosections were incubated with the appropriate secondary fluorescent antibodies. As a control, primary antibodies were omitted. Cryosections were visualized by a fluorescence Zeiss Axioscope Nikon digital DXM 1200. Digital images were collected using a Nikon Act-1 Version 2.10 software package. Digital images were processed using Confocal Assistant 4.02.

Reference List

1. Powers SK, Shanely RA, Coombes JS, Koesterer TJ, McKenzie M, Van Gammeren D et al. (2002) Mechanical ventilation results in progressive contractile dysfunction in the diaphragm. J Appl Physiol 92:1851-1858

2. Zuurbier CJ, Heslinga JW, Lee-de Groot MB, van der Laarse WJ (1995) Mean sarcomere length-force relationship of rat muscle fibre bundles. J Biomech 28:83-87

3. Burkholder TJ, Lieber RL (2001) Sarcomere length operating range of vertebrate muscles during movement. J Exp Biol 204:1529-1536

4. Ottenheijm CA, Heunks LM, Hafmans T, van der Ven PF, Benoist C, Zhou H et al. (2006) Titin and diaphragm dysfunction in chronic obstructive pulmonary disease. Am J Respir Crit Care Med 173:527-534

5. Wang K, McCarter R, Wright J, Beverly J, Ramirez-Mitchell R (1991) Regulation of skeletal muscle stiffness and elasticity by titin isoforms: a test of the segmental extension model of resting tension. Proc Natl Acad Sci U S A 88:7101-7105

6. Oakley BR, Kirsch DR, Morris NR (1980) A simplified ultrasensitive silver stain for detecting proteins in polyacrylamide gels. Anal Biochem 105:361-363

**Figure legends**

**Figure 1**

**A.** Silver stained SDS-polyacrylamide gel loaded with myosin standards, seven rat diaphragm single fibers and a homogenate of a rat diaphragm bundle.

**B.** Optical densities of myosin bands plotted against myosin content in the standards. Myosin content in the single fibers was derived from this standard curve.

**Figure 2**

Representative photographs of cross-sections of diaphragm from a control and mechanically ventilated rat stained with toluidine blue. Note the generally smaller size of fibers and larger distance between fibers in the diaphragm of the mechanically ventilated rat compared to control. Bar = 50µm.

**Figure 3**

Representative gel loaded with equal amounts of diaphragm homogenate. To discern size differences, the middle lane is loaded with homogenized human soleus containing titin of ~3700 kDa. T2 = titin degradation product.

Figure 1


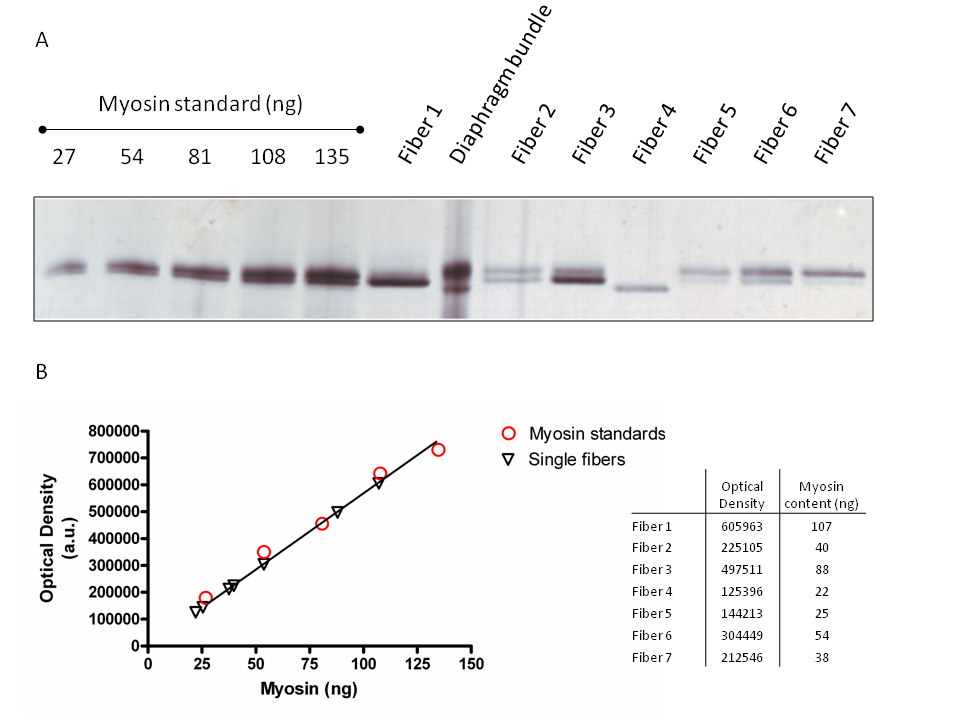


Figure 2


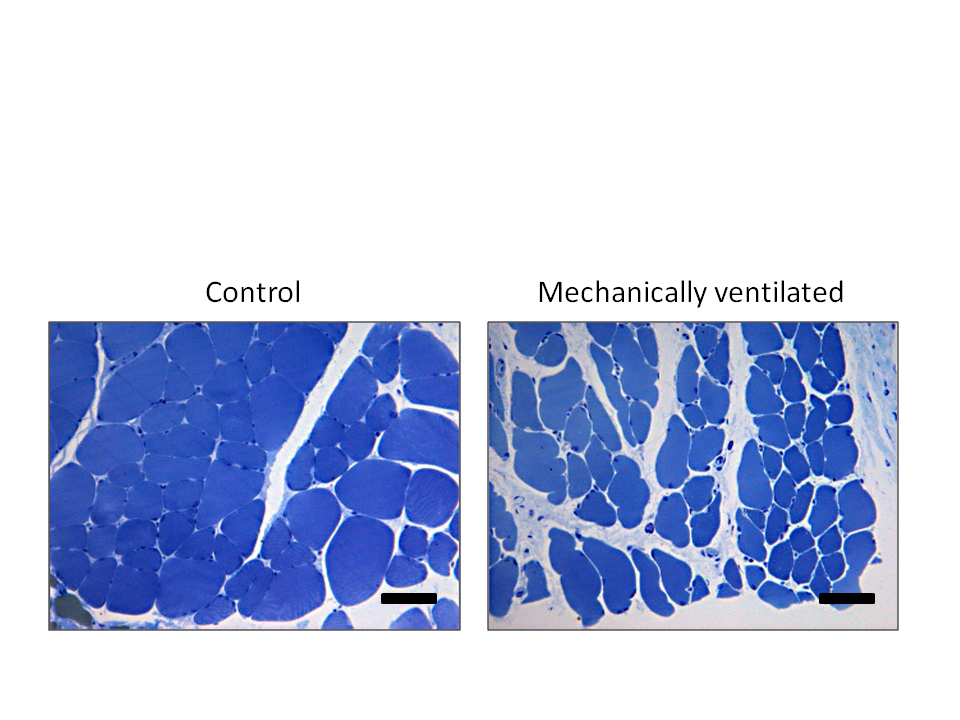


Figure 3


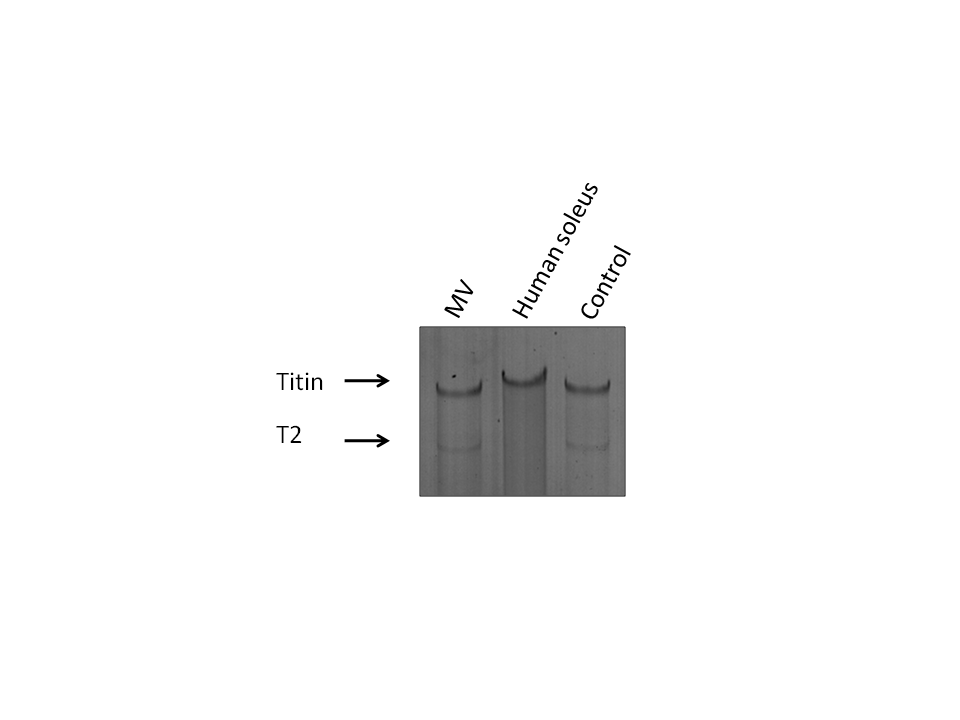

Supplement: Supplementary file 1 — Supplementary material 1 (DOC 1123 kb) [file 134_2012_2504_MOESM1_ESM.doc]
